# Three Children at One Birth

**Published:** 1895-12

**Authors:** C. Ferdinand Durand

**Affiliations:** Lockpokt, N. Y.


### THREE CHILDREN AT ONE BIRTH.

*Editor Buffalo Medical Journal:*

SIR—The following account of three children at a birth may prove interesting on account of its infrequency:

October 20, 1895, I was called to attend Mrs. R. in her fourth labor. Her abdominal distention was greater than is usual, but was not so markedly increased as to suggest what was to follow. Labor pains were constant all day, but the contractions lacked that propulsive character so often observed, no doubt due to over-distention of the uterus. The head presented in first position, and although the os was widely dilated, the uterine contractions were so feeble that natural delivery appeared impossible, so applying the forceps I delivered her of a boy. After cutting the cord I waited, as is my custom, for the placenta to be expelled, making pressure over the fundus. After waiting half an hour with the pains practically absent, I introduced my hand to deliver the placenta, when I felt the feet of another child through its amniotic sac. By traction on the feet I aided the descent of a second child, ruptured the membranes, and pains still being absent, I delivered the woman with what assistance she could give by voluntarily compressing the abdominal muscles.

After again waiting twenty minutes and making pressure through the abdomen as before, I introduced my hand into the uterus again and encountered through the walls of a third sac the breech of a third child. This also was delivered largely by the voluntary bearing down of the mother, though the uterine contractions seemed at the time to increase in power. The child's back was anterior. Within fifteen minutes of the birth of the last child, the uterus emptied itself. There were three sacs and three distinct placentas. There was neither subsequent hemorrhage nor rise of temperature, and the mother got up on the ninth day after labor. The first and second children were boys, the third a girl. When two days old the girl weighed four and one-half pounds, the first boy five and one-half pounds and the second boy six pounds, and up to the present time are alive and doing well.

The parents are both Germans. The father is thirty-five years old, medium-sized, dark-complexioned, and was a twin. The mother is twenty-six years old, over the average height and fair. Her first labor, I understand, was difficult, instruments having to be used. I attended her when she had her second child, a girl with talipes equino-varus of both feet. Her third child, a boy, lived only two days, although apparently strong when born.

C. FERDINAND DURAND.

LOCKPORT, N. Y.

